# Tribarium dicitrate penta­hydrate, [Ba_3_(C_6_H_5_O_7_)_2_(H_2_O)_4_]·H_2_O

**DOI:** 10.1107/S2056989021001407

**Published:** 2021-02-12

**Authors:** James A. Kaduk

**Affiliations:** a Department of Physics, North Central College, 131 S. Loomis St., Naperville IL 60540 , USA; b Department of Chemistry, Illinois Institute of Technology, 3101 S. Dearborn St., Chicago IL 60616 , USA

**Keywords:** powder diffraction, citrate, barium, Rietveld refinement, density functional theory

## Abstract

The crystal structure of tribarium dicitrate penta­hydrate, [Ba_3_(C_6_H_5_O_7_)_2_(H_2_O)_4_]·H_2_O, has been solved and refined using synchrotron X-ray powder diffraction data, and optimized using density functional techniques. The BaO_9_ and BaO_10_ coordination polyhedra share edges and corners to form a three-dimensional network. All of the active hydrogen atoms act as donors in O—H⋯O hydrogen bonds. Most of the acceptors are carboxyl­ate oxygen atoms, but there are also water⋯water hydrogen bonds. Both of the citrate hydroxyl groups form intra­molecular O—H⋯O hydrogen bonds to terminal carboxyl groups.

## Abstract 2

The crystal structure of tribarium dicitrate penta­hydrate, [Ba_3_(C_6_H_5_O_7_)_2_(H_2_O)_4_](H_2_O), has been solved and refined using synchrotron X-ray powder diffraction data, and optimized using density functional techniques.

## Chemical context   

A systematic study of the crystal structures of Group 1 (alkali metal) citrate salts has been reported in Rammohan & Kaduk (2018 ▸). The study was extended to mixed Group 1 citrates and to alkali/ammonium citrates in a series of papers, to magnesium citrates in Kaduk (2020*a* ▸), and to calcium citrates in Kaduk (2018 ▸) and Kaduk (2020*b* ▸). This paper represents a further extension to barium citrates and describes the synthesis and structure of the title compound, (I).
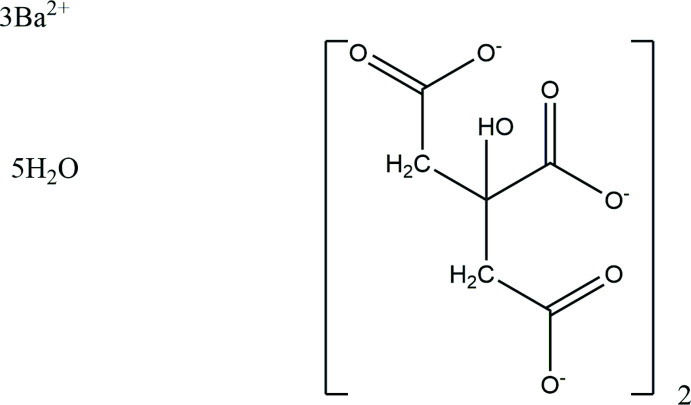


## Structural commentary   

**Figure 1:**
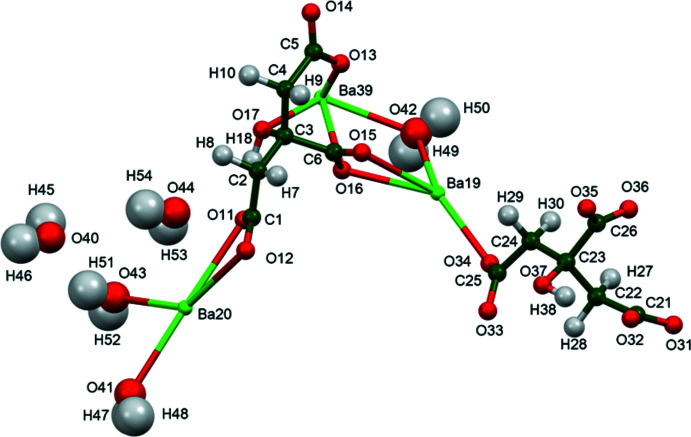
The asymmetric unit of (I) with the atom numbering and 50% probability spheres.

**Figure 2:**
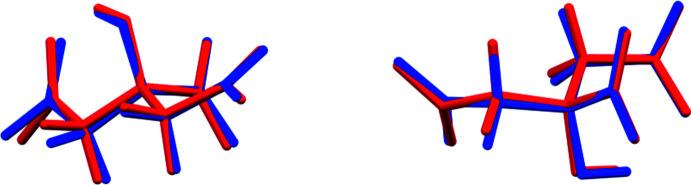
Comparison of the refined and optimized structures of the citrate anions in (I). The refined structure is in red, and the DFT-optimized structure is in blue. Citrate ion 1 (C1–H18) is on the left, and citrate ion 2 (C21–H38) is on the right.

The crystal structure of tribarium dicitrate penta­hydrate, [Ba_3_(C_6_H_5_O_7_)_2_(H_2_O)_4_](H_2_O), has been solved and refined using synchrotron X-ray powder diffraction data, and optimized using density functional techniques (Fig. 1 ▸). The root-mean-square Cartesian displacements of the non-H atoms in the Rietveld-refined and DFT-optimized structures of the two crystallographically distinct citrate anions are 0.155 and 0.093 Å (Fig. 2 ▸). The absolute differences in the positions of the three unique Ba^2+^ cations are 0.075, 0.345, and 0.081 Å. The good agreement between the structures is evidence that the experimental structure is correct (van de Streek & Neumann, 2014 ▸). The rest of the discussion will emphasize the DFT-optimized structure. Almost all of the citrate bond distances, bond angles, and torsion angles fall within the normal ranges indicated by a *Mercury* Mogul Geometry Check (Macrae *et al.*, 2020 ▸). The O13—C5—O14 bond angle of 122.1° is flagged as unusual [average = 123.8 (4)°, *Z*-score = 3.3]. The standard uncertainty on this average is exceptionally-small, inflating the *Z*-score. The C22—C23—C24—C25 torsion angle is flagged as unusual; it lies on the tail of a minor *gauche* population in a mainly *trans* distribution of similar torsion angles. Citrate anion 1 (atoms C1–H18) occurs in the *trans,trans*-conformation (about C2—C3 and C3—C4), which is one of the two low-energy conformations of an isolated citrate anion (Rammohan & Kaduk, 2018 ▸), while citrate anion 2 (C21–H38) is the in the *trans, gauche* conformation, which is the other low-energy arrangement. For the larger Group 1 cations, the *trans,trans* conformation is more typical. The central carboxyl­ate groups and the hydroxyl groups exhibit significant twists of −20 and −24° from the normal planar arrangement.

The three barium cations Ba19, Ba20, and Ba39 are ten-, nine- and ten-coordinate, respectively. Ba19 is coordinated to one water mol­ecule, eight carboxyl­ate oxygen atoms and one hydroxyl group. Ba20 is coordinated to three water mol­ecules and six carboxyl­ate oxygen atoms. Ba39 is coordinated to one water mol­ecule, seven carboxyl­ate oxygen atoms and two hydroxyl groups. Water mol­ecule O40 is uncoordinated. The bond-valence sums (in valence units) for Ba19, Ba20 and Ba39 are 2.20, 2.15 and 2.20, respectively. The Mulliken overlap populations indicate that the Ba—O bonds are ionic. Citrate anion 1 triply chelates to Ba19 through the terminal carboxyl­ate atom O13, the central carboxyl­ate O16 and the hydroxyl group O17. It doubly chelates to another Ba19 cation through the terminal carboxyl­ate O11 and the central carboxyl­ate O16. The terminal carboxyl­ate O13/O14 chelates to a third Ba19, and the central carboxyl­ate O15/O16 chelates to a fourth Ba19. Citrate 1 also chelates to Ba39 through the terminal carboxyl­ate O13 and the hydroxyl group O17. Citrate 2 chelates to Ba39 through the terminal carboxyl­ate O33 and the hydroxyl group O37. The terminal carboxyl­ate O33/O34 chelates to Ba39, the terminal carboxyl­ate O31/O32 chelates to Ba20 and the central carboxyl­ate O35/O36 chelates to another Ba20 cation.

## Supra­molecular features   

**Figure 3:**
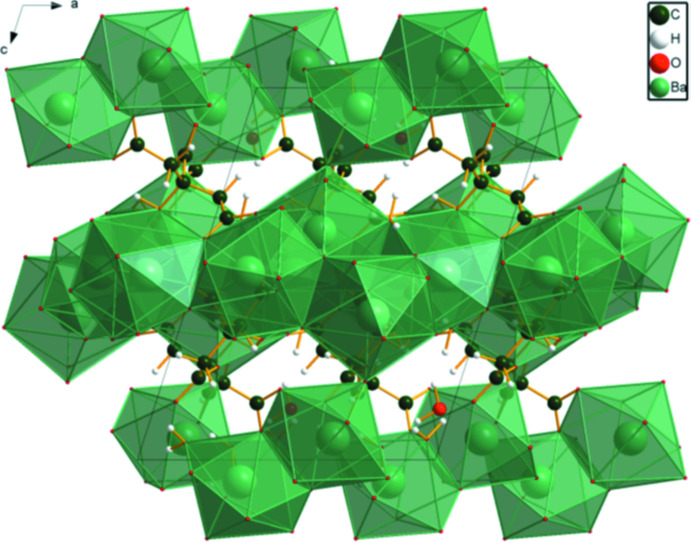
The crystal structure of (I), viewed down the *b*-axis direction.

The Ba coordination polyhedra share edges and corners to form a three-dimensional framework (Fig. 3 ▸). The framework contains edge-sharing layers propagating in the *ab* plane. These layers share corners to form the framework. All of the active hydrogen atoms act as donors in O—H⋯O hydrogen bonds: most of the acceptors are carboxyl­ate oxygen atoms, but there are also water⋯water hydrogen bonds (Table 1 ▸). Both of the hydroxyl groups form intra­molecular hydrogen bonds to terminal carboxyl groups. Two weak C—H⋯O hydrogen bonds also contribute to the packing.

## Database survey   

Details of the comprehensive literature search for citrate structures are presented in Rammohan & Kaduk (2018 ▸). A search of the Cambridge Structural Database (CSD, version 2020.3.0 from Dec 2020; Groom *et al.*, 2016 ▸) using a citrate fragment and the elements Ba, C, H, and O only yielded [Ba_5_(C_6_H_5_O_7_)_2_(HC_6_H_5_O_7_)_2_(H_2_O)_6_)](H_2_O)_2_ (Drzewiecka-Antonik *et al.*, 2017 ▸; refcode QASXAM), the structure of which was also determined independently (Kaduk & Mueller, 2020 ▸). A search of the Powder Diffraction File (Gates-Rector & Blanton, 2019 ▸) for barium citrates yielded only entry 00-001-0009 for barium citrate hepta­hydrate (Hanawalt *et al.*, 1938 ▸), one of the compounds in the first group of entries in the PDF. This powder pattern differs from that of the current compound.

## Synthesis and crystallization   

Tribarium dicitrate penta­hydrate was synthesized by dissolving 2.0818 g (10.0 mmol) of citric acid monohydrate in 25 ml of water, and adding 2.9615 g (15.0 mmol) of BaCO_3_ to the clear solution. After slow fizzing, some solid remained, so the slurry was heated to boiling, and additional fizzing occurred. The slurry was filtered and dried at room temperature to yield the title compound as a white powder.

## Refinement   

**Figure 4:**
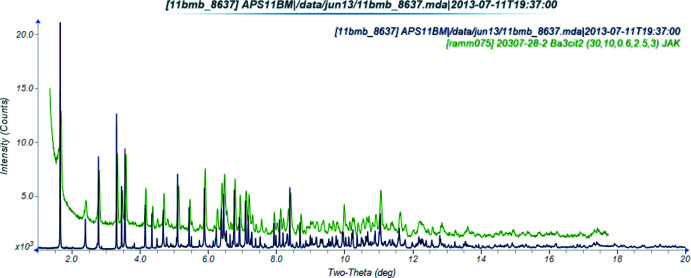
Comparison of the synchrotron (black) and laboratory X-ray powder diffraction patterns of (I). The laboratory pattern (measured using Cu *K*α radiation) was converted to the synchrotron wavelength of 0.413891 Å using *JADE Pro* (MDI, 2020 ▸).

Crystal data, data collection and structure refinement details are summarized in Table 2 ▸. A laboratory pattern, measured using Cu *K*α radiation, was indexed using *DICVOL06* (Louër & Boultif, 2007 ▸) on a primitive monoclinic cell with *a* = 11.4741, *b* = 13.7366, *c* = 15.0626 Å, *β* = 107.944°, *V* = 2258.62 Å^3^, and *Z* = 4. After attempts to solve the structure using the laboratory data were unsuccessful, the powder pattern was measured at beamline 11-BM at the Advanced Photon Source, Argonne National Laboratory using a wavelength of 0.413891 Å and was indexed on a similar cell (Fig. 4 ▸). The structure was solved using Monte Carlo simulated annealing techniques as implemented in *DASH* (David *et al.*, 2006 ▸). Three Ba atoms and two citrate anions were used as fragments. Oxygen atoms of water mol­ecules were placed in voids located by *Mercury* (Macrae *et al.*, 2020 ▸). Approximate positions of the hydrogen atoms were determined by analysis of potential hydrogen-bonding patterns.

**Figure 5:**
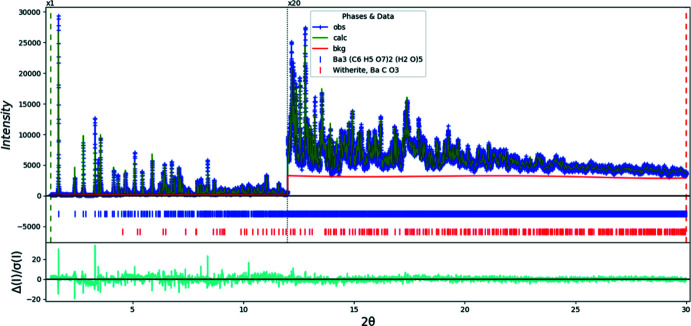
Rietveld plot for (I). The blue crosses represent the observed data points, and the green line is the calculated pattern. The cyan curve is the normalized error plot. The vertical scale has been multiplied by a factor of 20× for 2θ > 12.0°. The row of blue tick marks indicates the calculated reflection positions, and the red tick marks indicate the peak positions for the BaCO_3_ impurity. The red line is the background curve.

The structure was refined by the Rietveld method using *GSAS-II* (Toby & Von Dreele, 2013 ▸). The initial refinement clarified the presence of extra peaks, which were identified as witherite, BaCO_3_, which was added as a second phase; its contribution refined to 9.2 wt%. All non-H bond distances and angles in the citrate anions were subjected to restraints, based on a *Mercury* Mogul Geometry Check (Sykes *et al.*, 2011 ▸; Bruno *et al.*, 2004 ▸); the Ba—O distances were not restrained. The Mogul average and standard deviation for each qu­antity were used as the restraint parameters. The restraints contributed 1.5% to the final *χ* ^2^. The hydrogen atoms were included in calculated positions, which were recalculated during the refinement using *Materials Studio* (Dassault Systems, 2020 ▸). The *U*_iso_ values (Å^2^) were grouped by chemical similarity; the *U*_iso_ for the H atoms were fixed at 1.3 × the *U*_iso_ of the heavy atoms to which they are attached. Attempts to refine the *U*_iso_ of the C and O atoms of the citrate anions led to values very close to zero, so these were fixed at reasonable values based on experience. The generalized microstrain model was used to describe the peak profiles. A 4th-order spherical harmonics preferred orientation model was included; the texture index refined to 1.006. The background was described by a six-term shifted Chebyshev polynomial, with a peak at 5.60° to describe the scattering from the Kapton capillary and any amorphous component. The largest errors in the fit (Fig. 5 ▸) are in the positions and shapes of some of the strong low-angle peaks, and suggest that the specimen changed during exposure to the X-ray beam.

A density functional geometry optimization (fixed experimental unit cell) was carried out using *CRYSTAL09* (Dovesi *et al.*, 2018 ▸). The basis sets for the H, C and O atoms were those of Gatti *et al.* (1994 ▸), and the basis set for Ba was that of Piskunov *et al.* (2004 ▸). The calculation used 8 *k*-points and the B3LYP functional, and took ∼10.5 days on a 2.4 GHz PC.

## Supplementary Material

Crystal structure: contains datablock(s) global, I_DFT, hb7964_overall, I, II, ramm075_8637_pwd_0. DOI: 10.1107/S2056989021001407/hb7964sup1.cif

CCDC references: 2061757, 2061758, 2061759

Additional supporting information:  crystallographic information; 3D view; checkCIF report

## Figures and Tables

**Table 1:** Hydrogen-bond geometry (Å, °)

*D*—H⋯*A*	*D*—H	H⋯*A*	*D*⋯*A*	*D*—H⋯*A*
O17—H18⋯O11	0.99	1.80	2.675	146
O37—H38⋯O32	0.98	1.90	2.742	142
O40—H45⋯O44^i^	0.97	1.94	2.862	156
O40—H46⋯O34^ii^	0.97	2.01	2.959	165
O41—H47⋯O12^iii^	0.98	1.78	2.718	159
O41—H48⋯O44^i^	0.97	2.43	3.257	143
O42—H49⋯O14^iii^	0.98	1.75	2.629	147
O42—H50⋯O32^ii^	0.99	1.68	2.642	164
O43—H51⋯O40	0.99	1.73	2.711	171
O43—H52⋯O41	0.97	2.07	2.963	151
O44—H53⋯O12^iv^	0.97	1.94	2.804	146
O44—H54⋯O36^v^	0.98	1.80	2.707	152
C4—H9⋯O33^vi^	1.09	2.42	3.411	150
C22—H27⋯O40^ii^	1.09	2.54	3.534	151

Symmetry codes: (i) -x+{\script{3\over 2}}, y-{\script{1\over 2}}, -z+1; (ii) -x+1, -y+1, -z+1; (iii) x+{\script{1\over 2}}, -y+{\script{1\over 2}}, z; (iv) -x+{\script{1\over 2}}, y+{\script{1\over 2}}, -z+1; (v) -x+{\script{3\over 2}}, y+{\script{1\over 2}}, -z+2; (vi) -x+{\script{1\over 2}}, y-{\script{1\over 2}}, -z+1.

**Table 2:** Experimental details

Crystal data	
Chemical formula	[Ba_3_(C_6_H_5_O_7_)_2_(H_2_O)_4_]·H_2_O
*M*_r_	880.26
Crystal system, space group	Monoclinic, *P*2_1_/*a*
Temperature (K)	295
*a*, *b*, *c* (Å)	11.4768 (2), 13.75186 (7), 15.0943 (4)
β (°)	107.7746 (7)
*V* (Å^3^)	2268.57 (2)
*Z*	4
Radiation type	Synchrotron, λ = 0.41389 Å
μ (mm^−1^)	0.57
Specimen shape, size (mm)	Cylinder, 3.0 × 1.5

Data collection	
Diffractometer	11-BM, APS
Specimen mounting	Kapton capillary
Data collection mode	Transmission
Scan method	Step
2θ values (°)	2θ_min_ = 0.500, 2θ_max_ = 49.994, 2θ_step_ = 0.001

Refinement	
*R* factors and goodness of fit	*R*_p_ = 0.105, *R*_wp_ = 0.111, *R*_exp_ = 0.050, χ^2^ = 4.995
No. of parameters	133
No. of restraints	58
(Δ/σ)_max_	3.844

Computer programs: *DASH* (David *et al.*, 2006 ▸), *GSAS-II* (Toby & Von Dreele, 2013 ▸), *Mercury* (Macrae *et al.*, 2020 ▸), *DIAMOND* (Crystal Impact, 2015 ▸), and *publCIF* (Westrip, 2010 ▸).

## supplementary crystallographic information

### Tribarium dicitrate pentahydrate (I) Crystal data

**Table:**

[Ba_3_(C_6_H_5_O_7_)_2_(H_2_O)_4_]·H_2_O	*V* = 2268.57 (2) Å^3^
*M**_r_* = 880.26	*Z* = 4
Monoclinic, *P*2_1_/*a*	*D*_x_ = 2.577 Mg m^−^^3^
Hall symbol: -P 2yab	Synchrotron radiation
*a* = 11.4768 (2) Å	µ = 0.57 mm^−^^1^
*b* = 13.75186 (7) Å	*T* = 295 K
*c* = 15.0943 (4) Å	cylinder, 3.0 × 1.5 mm
β = 107.7746 (7)°	

### Tribarium dicitrate pentahydrate (I) Data collection

**Table:**

11-BM, APS diffractometer	Data collection mode: transmission
Specimen mounting: Kapton capillary	Scan method: step

### Tribarium dicitrate pentahydrate (I) Refinement

**Table:**

Profile function: Crystallite size in microns with "isotropic" model: parameters: Size, G/L mix 1.000, 1.000, Microstrain, "generalized" model (10^6^ * delta Q/Q) parameters: S400, S040, S004, S220, S202, S022, S301, S103, S121, G/L mix 335.804, 65.612, 324.354, 213.281, 392.656, 323.823, 45.812, 408.669, 280.544, 1.000,	Preferred orientation correction: Simple spherical harmonic correction Order = 4 Coefficients: 0:0:C(2,-2) = -0.042(4); 0:0:C(2,0) = 0.073(7); 0:0:C(2,2) = -0.088(5); 0:0:C(4,-4) = -0.024(7); 0:0:C(4,-2) = 0.001(6); 0:0:C(4,0) = 0.156(8); 0:0:C(4,2) = -0.061(6); 0:0:C(4,4) = 0.020(7)

### Tribarium dicitrate pentahydrate (I) Fractional atomic coordinates and isotropic or equivalent isotropic displacement parameters (Å^2^)

**Table:**

	*x*	*y*	*z*	*U*_iso_*/*U*_eq_	
C1	−0.1025 (14)	0.3842 (7)	0.2342 (11)	0.030*	
C2	−0.1218 (17)	0.2808 (7)	0.2617 (10)	0.030*	
C3	−0.1696 (9)	0.2738 (5)	0.3463 (6)	0.030000*	
C4	−0.1944 (15)	0.1670 (6)	0.3637 (8)	0.030000*	
C5	−0.234 (2)	0.1472 (9)	0.4497 (10)	0.030000*	
C6	−0.0758 (11)	0.3156 (8)	0.4336 (9)	0.030000*	
H7	−0.03119	0.24004	0.27815	0.039*	
H8	−0.19153	0.24301	0.20112	0.039*	
H9	−0.10789	0.12334	0.36968	0.039*	
H10	−0.27095	0.13789	0.30201	0.039*	
O11	−0.1735 (14)	0.4478 (9)	0.2382 (12)	0.030000*	
O12	−0.0273 (13)	0.3949 (9)	0.1903 (11)	0.030000*	
O13	−0.2195 (16)	0.2142 (10)	0.5088 (10)	0.030000*	
O14	−0.2699 (15)	0.0654 (9)	0.4606 (10)	0.030000*	
O15	0.0211 (11)	0.2702 (11)	0.4695 (11)	0.030000*	
O16	−0.0950 (13)	0.3999 (9)	0.4574 (11)	0.030000*	
O17	−0.2813 (10)	0.3277 (9)	0.3297 (9)	0.030000*	
H18	−0.27014	0.38695	0.29865	0.039*	
Ba19	0.13690 (18)	0.40567 (15)	0.61015 (14)	0.0206 (3)*	
Ba20	−0.13661 (19)	0.56012 (14)	0.07100 (16)	0.0206*	
C21	0.8413 (9)	0.4134 (14)	0.8464 (12)	0.030000*	
C22	0.7053 (10)	0.4247 (7)	0.7965 (11)	0.030000*	
C23	0.6437 (8)	0.3321 (6)	0.7475 (6)	0.030000*	
C24	0.5045 (8)	0.3466 (9)	0.7101 (10)	0.030000*	
C25	0.4557 (11)	0.4271 (9)	0.6402 (9)	0.030000*	
C26	0.6710 (16)	0.2470 (7)	0.8181 (7)	0.030000*	
H27	0.65616	0.44600	0.84882	0.039*	
H28	0.69212	0.48515	0.74264	0.039*	
H29	0.45979	0.27739	0.67362	0.039*	
H30	0.46933	0.35967	0.77203	0.039*	
O31	0.8872 (12)	0.4619 (12)	0.9199 (9)	0.030000*	
O32	0.9015 (12)	0.3548 (11)	0.8130 (11)	0.030000*	
O33	0.5172 (14)	0.4590 (11)	0.5921 (12)	0.030000*	
O34	0.3680 (12)	0.4732 (10)	0.6521 (12)	0.030000*	
O35	0.6922 (17)	0.1668 (8)	0.7892 (10)	0.030000*	
O36	0.6695 (17)	0.2652 (10)	0.8982 (8)	0.030000*	
O37	0.6907 (13)	0.3101 (9)	0.6718 (9)	0.030000*	
H38	0.7669	0.3079	0.6998	0.039*	
Ba39	−0.33612 (17)	0.39258 (15)	0.49603 (15)	0.0206*	
O40	0.532 (2)	0.4922 (15)	0.1197 (15)	0.088 (4)*	
O41	0.601 (2)	0.1767 (15)	0.0858 (18)	0.088*	
O42	0.0959 (19)	0.5739 (16)	0.3574 (17)	0.088*	
O43	0.375 (2)	0.3603 (14)	0.0222 (17)	0.088*	
O44	0.676 (2)	0.8072 (15)	0.9286 (18)	0.088*	
H45	0.60087	0.47209	0.10408	0.144*	
H46	0.5683	0.4887	0.1778	0.144*	
H47	0.5350	0.1822	0.1001	0.144*	
H48	0.6000	0.2384	0.0855	0.144*	
H49	0.11724	0.51249	0.37158	0.144*	
H50	0.0844	0.58877	0.30093	0.144*	
H51	0.4122	0.3900	0.0720	0.144*	
H52	0.40460	0.30290	0.02846	0.144*	
H53	0.7131	0.8684	0.9342	0.144*	
H54	0.7236	0.7942	0.9823	0.144*	

### Tribarium dicitrate pentahydrate (I) Geometric parameters (Å, º)

**Table:**

C1—C2	1.515 (4)	Ba20—O43^ix^	2.88 (2)
C1—O11	1.210 (6)	C21—C22	1.520 (4)
C1—O12	1.246 (4)	C21—O31	1.263 (5)
C2—C1	1.515 (4)	C21—O32	1.262 (5)
C2—C3	1.538 (3)	C22—C21	1.520 (4)
C3—C2	1.538 (3)	C22—C23	1.533 (3)
C3—C4	1.5334 (11)	C23—C22	1.533 (3)
C3—C6	1.537 (2)	C23—C24	1.537 (3)
C3—O17	1.435 (3)	C23—C26	1.549 (3)
C4—C3	1.5334 (11)	C23—O37	1.436 (4)
C4—C5	1.523 (6)	C24—C23	1.537 (3)
C5—C4	1.523 (6)	C24—C25	1.514 (4)
C5—O13	1.259 (9)	C25—C24	1.514 (4)
C5—O14	1.228 (6)	C25—O33	1.237 (6)
C6—C3	1.537 (2)	C25—O34	1.248 (4)
C6—O15	1.246 (4)	C26—C23	1.549 (3)
C6—O16	1.253 (3)	C26—O35	1.236 (7)
O11—C1	1.210 (6)	C26—O36	1.240 (7)
O11—Ba19^i^	2.982 (16)	O31—Ba20^x^	2.736 (13)
O12—C1	1.246 (4)	O31—Ba20^vi^	2.840 (15)
O12—Ba20	2.933 (14)	O31—C21	1.263 (5)
O13—C5	1.259 (9)	O32—Ba20^vi^	2.971 (15)
O13—Ba39	2.772 (16)	O32—C21	1.262 (5)
O14—C5	1.228 (6)	O33—C25	1.237 (6)
O14—Ba19^ii^	2.805 (13)	O33—Ba39^xi^	2.695 (14)
O14—Ba39^iii^	2.660 (15)	O33—Ba39^i^	2.927 (14)
O15—C6	1.246 (4)	O34—Ba19	2.697 (15)
O15—Ba19	2.833 (14)	O34—C25	1.248 (4)
O15—Ba39^iv^	2.730 (13)	O34—Ba39^i^	2.835 (14)
O16—C6	1.253 (3)	O35—Ba19^iv^	2.767 (14)
O16—Ba19	2.942 (13)	O35—Ba20^xii^	2.799 (14)
O16—Ba19^i^	2.850 (15)	O35—C26	1.236 (7)
O16—Ba39	2.999 (16)	O36—Ba20^xii^	2.901 (13)
O17—C3	1.435 (3)	O36—C26	1.240 (7)
O17—Ba39	2.909 (13)	O37—C23	1.436 (4)
Ba19—O11^i^	2.982 (16)	O37—Ba39^xi^	2.815 (12)
Ba19—O14^iv^	2.805 (13)	Ba39—O13	2.772 (16)
Ba19—O15	2.833 (14)	Ba39—O14^xiii^	2.660 (15)
Ba19—O16	2.942 (13)	Ba39—O15^ii^	2.730 (13)
Ba19—O16^i^	2.850 (15)	Ba39—O16	2.999 (16)
Ba19—O34	2.697 (15)	Ba39—O17	2.909 (13)
Ba19—O35^ii^	2.767 (14)	Ba39—O33^xiv^	2.695 (14)
Ba19—O42^i^	2.87 (2)	Ba39—O33^i^	2.927 (14)
Ba20—O12	2.933 (14)	Ba39—O34^i^	2.835 (14)
Ba20—O31^v^	2.736 (13)	Ba39—O37^xiv^	2.815 (12)
Ba20—O31^vi^	2.840 (15)	Ba39—O42^i^	3.00 (2)
Ba20—O32^vi^	2.971 (15)	O41—Ba20^xv^	2.99 (2)
Ba20—O35^vii^	2.799 (14)	O42—Ba19^i^	2.87 (2)
Ba20—O36^vii^	2.901 (13)	O42—Ba39^i^	3.00 (2)
Ba20—O41^viii^	2.99 (2)	O43—Ba20^ix^	2.88 (2)
			
C2—C1—O11	120.5 (6)	C22—C21—O31	118.1 (5)
C2—C1—O12	116.1 (3)	C22—C21—O32	118.2 (5)
O11—C1—O12	121.8 (5)	O31—C21—O32	123.7 (5)
C1—C2—C3	114.0 (4)	C21—C22—C23	114.0 (5)
C2—C3—C4	109.5 (2)	C22—C23—C24	110.1 (5)
C2—C3—C6	110.7 (2)	C22—C23—C26	109.2 (4)
C4—C3—C6	109.3 (2)	C24—C23—C26	108.7 (5)
C2—C3—O17	110.4 (2)	C22—C23—O37	109.2 (4)
C4—C3—O17	108.8 (3)	C24—C23—O37	109.6 (4)
C6—C3—O17	108.2 (3)	C26—C23—O37	110.0 (4)
C3—C4—C5	115.8 (2)	C23—C24—C25	118.3 (5)
C4—C5—O13	117.8 (3)	C24—C25—O33	120.8 (5)
C4—C5—O14	118.6 (4)	C24—C25—O34	114.5 (4)
O13—C5—O14	123.44 (11)	O33—C25—O34	122.3 (5)
C3—C6—O15	119.0 (4)	C23—C26—O35	116.8 (5)
C3—C6—O16	117.0 (5)	C23—C26—O36	117.3 (3)
O15—C6—O16	123.4 (7)	O35—C26—O36	125.9 (5)
C5—O14—Ba39^iii^	134.7 (18)		

Symmetry codes: (i) −*x*, −*y*+1, −*z*+1; (ii) *x*−1/2, −*y*+1/2, *z*; (iii) −*x*−1/2, *y*−1/2, −*z*+1; (iv) *x*+1/2, −*y*+1/2, *z*; (v) *x*−1, *y*, *z*−1; (vi) −*x*+1, −*y*+1, −*z*+1; (vii) −*x*+1/2, *y*+1/2, −*z*+1; (viii) −*x*+1/2, *y*+1/2, −*z*; (ix) −*x*, −*y*+1, −*z*; (x) *x*+1, *y*, *z*+1; (xi) *x*+1, *y*, *z*; (xii) −*x*+1/2, *y*−1/2, −*z*+1; (xiii) −*x*−1/2, *y*+1/2, −*z*+1; (xiv) *x*−1, *y*, *z*; (xv) −*x*+1/2, *y*−1/2, −*z*.

### Barium carbonate (II) Crystal data

**Table:**

Ba^2+^·CO_3_^2^^−^	*V* = 304.24 Å^3^
*M**_r_* = 197.34	*Z* = 4
Orthorhombic, *P**m**c**n*	*D*_x_ = 4.308 Mg m^−^^3^
Hall symbol: -P 2n 2a	Synchrotron radiation
*a* = 5.307826 Å	*T* = 295 K
*b* = 8.91479 Å	cylinder, 3.0 × 1.5 mm
*c* = 6.429736 Å	

### Barium carbonate (II) Data collection

**Table:**

11-BM, APS diffractometer	Data collection mode: transmission
Specimen mounting: Kapton capillary	Scan method: step

### Barium carbonate (II) Refinement

**Table:**

Profile function: Crystallite size in microns with "isotropic" model: parameters: Size, G/L mix 1.000, 1.000, Microstrain, "isotropic" model (10^6^ * delta Q/Q) parameters: Mustrain, G/L mix 6653.476, 1.000,	Preferred orientation correction: March-Dollase correction coef. = 1.000 axis = [0, 0, 1]

### Barium carbonate (II) Fractional atomic coordinates and isotropic or equivalent isotropic displacement parameters (Å^2^)

**Table:**

	*x*	*y*	*z*	*U*_iso_*/*U*_eq_	
Ba1	0.25000	0.41660	0.75300	0.004*	
C2	0.25000	0.75550	−0.08170	0.010*	
O3	0.25000	0.89990	−0.09230	0.010*	
O4	0.45960	0.68310	−0.07940	0.010*	

### (I_DFT) Crystal data

**Table:**

C12H20Ba3O_19_	*b* = 13.75185 Å
*M**_r_* = 880.26	*c* = 15.09415 Å
Monoclinic, *P*2_1_/*a*	β = 107.7751°
Hall symbol: -P 2yab	*V* = 2268.57 Å^3^
*a* = 11.47665 Å	*Z* = 4

### (I_DFT) Data collection

**Table:**

DFT calculation	*k* = →
*h* = →	*l* = →

### (I_DFT) Fractional atomic coordinates and isotropic or equivalent isotropic displacement parameters (Å^2^)

**Table:**

	*x*	*y*	*z*	*U*_iso_*/*U*_eq_	
C1	−0.11688	0.38341	0.22166	0.03000*	
C2	−0.11971	0.28083	0.26110	0.03000*	
C3	−0.17294	0.27375	0.34400	0.03000*	
C4	−0.19983	0.16788	0.36121	0.03000*	
C5	−0.23879	0.14905	0.44777	0.03000*	
C6	−0.07688	0.31797	0.43048	0.03000*	
H7	−0.02622	0.25203	0.28217	0.03000*	
H8	−0.17156	0.23439	0.20331	0.03000*	
H9	−0.11930	0.12249	0.36798	0.03000*	
H10	−0.27184	0.14085	0.30039	0.03000*	
O11	−0.16833	0.45272	0.24996	0.03000*	
O12	−0.06710	0.39334	0.15745	0.03000*	
O13	−0.22149	0.21126	0.51164	0.03000*	
O14	−0.28623	0.06598	0.45292	0.03000*	
O15	0.01583	0.26683	0.47045	0.03000*	
O16	−0.09383	0.40371	0.45517	0.03000*	
O17	−0.28664	0.32466	0.32474	0.03000*	
H18	−0.27014	0.38695	0.29865	0.03000*	
Ba19	0.13385	0.40167	0.61196	0.03000*	
Ba20	−0.13311	0.57982	0.05792	0.03000*	
C21	0.83987	0.41712	0.84661	0.03000*	
C22	0.70518	0.42971	0.79294	0.03000*	
C23	0.64524	0.33505	0.74625	0.03000*	
C24	0.50573	0.34541	0.70474	0.03000*	
C25	0.46045	0.43800	0.64958	0.03000*	
C26	0.66754	0.25160	0.81797	0.03000*	
H27	0.65561	0.45891	0.83816	0.03000*	
H28	0.69959	0.48306	0.73791	0.03000*	
H29	0.47098	0.28497	0.65661	0.03000*	
H30	0.46497	0.33972	0.76116	0.03000*	
O31	0.88519	0.46054	0.92241	0.03000*	
O32	0.90416	0.36375	0.80963	0.03000*	
O33	0.51325	0.46906	0.59293	0.03000*	
O34	0.36709	0.47967	0.66147	0.03000*	
O35	0.68183	0.16680	0.79036	0.03000*	
O36	0.66454	0.27127	0.89887	0.03000*	
O37	0.69630	0.30673	0.67385	0.03000*	
H38	0.78487	0.30732	0.70637	0.03000*	
Ba39	−0.32983	0.38942	0.49723	0.03000*	
O40	0.54019	0.47768	0.13700	0.03000*	
O41	0.55197	0.20809	0.05681	0.03000*	
O42	0.08949	0.57977	0.35613	0.03000*	
O43	0.35851	0.35404	0.04715	0.03000*	
O44	0.80523	0.90671	0.96587	0.03000*	
H45	0.60087	0.47209	0.10408	0.03000*	
H46	0.58408	0.48718	0.20266	0.03000*	
H47	0.51630	0.18379	0.10418	0.03000*	
H48	0.59957	0.26591	0.08079	0.03000*	
H49	0.11724	0.51249	0.37158	0.03000*	
H50	0.08221	0.59147	0.29023	0.03000*	
H51	0.42234	0.39813	0.08554	0.03000*	
H52	0.40460	0.30290	0.02846	0.03000*	
H53	0.72513	0.87814	0.93604	0.03000*	
H54	0.83408	0.87282	1.02599	0.03000*	

### (I_DFT) Bond lengths (Å)

**Table:**

C1—C2	1.536	C23—C26	1.544
C1—O11	1.263	C23—O37	1.442
C1—O12	1.274	C24—C25	1.525
C2—C3	1.552	C24—H29	1.096
C2—H7	1.096	C24—H30	1.092
C2—H8	1.098	C25—C24	1.525
C3—C4	1.528	C25—O33	1.265
C3—C6	1.553	C25—O34	1.275
C3—O17	1.430	C26—O35	1.265
C4—C5	1.526	C26—O36	1.255
C4—H9	1.094	H29—C24	1.096
C4—H10	1.096	H30—C24	1.092
C5—O13	1.258	O33—C25	1.265
C5—O14	1.278	O37—H38	0.980
C6—O15	1.264	O40—H45	0.968
C6—O16	1.271	O40—H46	0.973
O13—C5	1.258	O41—H47	0.981
O17—H18	0.977	O41—H48	0.974
C21—C22	1.520	O42—H49	0.983
C21—O31	1.255	O42—H50	0.985
C21—O32	1.283	O43—H51	0.986
C22—C23	1.539	O43—H52	0.972
C22—H27	1.090	O44—H53	0.973
C22—H28	1.095	O44—H54	0.983
C23—C24	1.539		

### (I_DFT) Hydrogen-bond geometry (Å, º)

**Table:**

*D*—H···*A*	*D*—H	H···*A*	*D*···*A*	*D*—H···*A*
O17—H18···O11	0.99	1.80	2.675	146
O37—H38···O32	0.98	1.90	2.742	142
O40—H45···O44^i^	0.97	1.94	2.862	156
O40—H46···O34^ii^	0.97	2.01	2.959	165
O41—H47···O12^iii^	0.98	1.78	2.718	159
O41—H48···O44^i^	0.97	2.43	3.257	143
O42—H49···O14^iii^	0.98	1.75	2.629	147
O42—H50···O32^ii^	0.99	1.68	2.642	164
O43—H51···O40	0.99	1.73	2.711	171
O43—H52···O41	0.97	2.07	2.963	151
O44—H53···O12^iv^	0.97	1.94	2.804	146
O44—H54···O36^v^	0.98	1.80	2.707	152
C4—H9···O33^vi^	1.09	2.42	3.411	150
C22—H27···O40^ii^	1.09	2.54	3.534	151

Symmetry codes: (i) −*x*+3/2, *y*−1/2, −*z*+1; (ii) −*x*+1, −*y*+1, −*z*+1; (iii) *x*+1/2, −*y*+1/2, *z*; (iv) −*x*+1/2, *y*+1/2, −*z*+1; (v) −*x*+3/2, *y*+1/2, −*z*+2; (vi) −*x*+1/2, *y*−1/2, −*z*+1.
